# Short-Chain Fatty Acids Enhance EAAT2-Mediated Glutamate Clearance and Alleviate Oxidative Stress in an MPTP Mouse Model of Parkinson’s Disease

**DOI:** 10.3390/antiox14121429

**Published:** 2025-11-27

**Authors:** Weiqi Li, Jiali Li, Lulu Liu, Wenzhe Hu, Lei Wu, Songtao Ding, Bin Yu, Lin Jiang, Handeng Liu

**Affiliations:** 1 Laboratory of Tissue and Cell Biology, Experimental Teaching and Management Center, Chongqing Medical University, Chongqing 400016, China; 2 College of First Clinical, Chongqing Medical University, Chongqing 400016, China; 3 International Medical College, Chongqing Medical University, Chongqing 400016, China; 4 College of Pediatrics, Chongqing Medical University, Chongqing 400016, China; 5 Key Laboratory of Major Brain Disease and Aging Research (Ministry of Education), Chongqing Medical University, Chongqing 400016, China; 6 Center for Neuroscience Research, Chongqing Medical University, Chongqing 400016, China

**Keywords:** Parkinson’s disease, short-chain fatty acids (SCFAs), EAAT2, oxidative stress, astrocyte

## Abstract

Parkinson’s disease (PD) is a progressive neurodegenerative disorder characterized by dopaminergic neuron loss, motor deficits, and oxidative stress. Emerging evidence suggests that short-chain fatty acids (SCFAs), microbial metabolites derived from gut fermentation, exert neuroprotective effects, but the underlying mechanisms remain incompletely understood. In this study, we investigated the role of SCFAs in modulating astrocytic glutamate clearance and oxidative stress in a PD mouse model induced by 1-methyl-4-phenyl-1,2,3,6-tetrahydropyridine (MPTP). Behavioral tests demonstrated that SCFA treatment significantly improved locomotor activity, grip strength, and coordination, while attenuating dopaminergic neuron loss and tyrosine hydroxylase (TH) reduction in the substantia nigra and striatum. Mechanistically, SCFAs enhanced astrocytic glutamate uptake mediated by excitatory amino acid transporter 2 (EAAT2), suppressed astrocyte reactivity, and reduced neuroinflammation, as evidenced by decreased plasma interleukin-6 (IL-6), interleukin-1 beta (IL-1β), and tumor necrosis factor-alpha (TNF-α) levels. SCFAs also restored redox homeostasis by elevating glutathione, reducing malondialdehyde, preserving superoxide dismutase activity, and promoting nuclear factor-erythroid 2–related factor 2 (Nrf2) nuclear translocation with upregulation of downstream antioxidant enzymes like heme oxygenase-1 (HO-1), superoxide dismutase 1 (SOD1), and superoxide dismutase 2 (SOD2). Inhibition of EAAT2 with dihydrokainic acid (DHK) abolished the beneficial effects of SCFAs, highlighting the critical role of EAAT2 in mediating SCFA-driven neuroprotection. Collectively, our findings demonstrate that SCFAs confer neuroprotection in PD by enhancing EAAT2-dependent glutamate clearance, reducing reactive oxygen species (ROS) accumulation, and activating Nrf2-dependent antioxidant pathways, providing a mechanistic basis for SCFA-based therapeutic strategies in PD.

## 1. Introduction

Parkinson’s disease (PD) is a progressive neurodegenerative disorder whose principal pathological features are the loss of dopaminergic neurons in the substantia nigra and the formation of Lewy bodies from abnormal aggregates of α-synuclein. Patients with PD commonly present with asymmetric motor symptoms, including bradykinesia, resting tremor, and gait disturbance, and frequently experience a range of non-motor symptoms such as autonomic dysfunction, sleep disorders, mood disturbances, and cognitive decline [1,2,3]. Although current pharmacotherapies such as levodopa can ameliorate some PD symptoms, they have substantial limitations in terms of long-term efficacy, improvement of non-motor symptoms, and reduction in side effects [4,5,6].

The pathogenesis of PD is multifactorial, involving α-synuclein aggregation, mitochondrial dysfunction, neuroinflammation, and impairments in protein degradation systems [7,8,9]. Increasing evidence indicates that oxidative stress is intimately associated with PD and constitutes an important component among these pathogenic factors [10,11,12]. Oxidative stress, principally via generation of reactive oxygen species (ROS), can cause mitochondrial DNA damage, promote intracerebral spread of α-synuclein, and even drive structural damage to the nucleus and mislocalization of key transcription factors, thereby accelerating neuronal injury and death [13,14,15]. Thus, controlling oxidative stress in PD has emerged as a critical therapeutic focus.

Short-chain fatty acids (SCFAs) are key microbial metabolites produced by gut microbiota fermentation of dietary fiber; the main SCFAs are acetate, propionate, and butyrate. Besides serving as local energy substrates in the gut, SCFAs modulate central nervous system function via neural, endocrine, and immune pathways [16]. In PD patients, characteristic alterations of the gut microbiota have been reported, notably decreased abundance of SCFA-producing bacteria (particularly those synthesizing propionate and butyrate) and reduced microbial diversity [17]. Increasing evidence indicates that SCFAs can inhibit inflammation-related pathways, thereby exerting neuroprotective effects in PD models [18]. Some reports further hypothesize that SCFAs attenuate α-synuclein aggregation–induced oxidative stress by promoting Nrf2 expression [19]. However, the precise mechanisms by which SCFAs regulate antioxidant defenses remain incompletely defined.

Glutamate (Glu) is the principal excitatory neurotransmitter in the brain and participates in multiple physiological processes. During synaptic transmission, glutamate is released from presynaptic neurons and binds to postsynaptic N-methyl-D-aspartate (NMDA), α-amino-3-hydroxy-5-methyl-4-isoxazolepropionic acid (AMPA), and metabotropic glutamate receptors (mGluRs) to convey excitatory signals [20]. Under physiological conditions, approximately 80% of extracellular glutamate clearance is mediated by the astrocytic glutamate transporter excitatory amino acid transporter 2 (EAAT2), which plays a central role in glutamate metabolism and transport [21]. Previous studies have indicated that SCFA treatment can upregulate EAAT2 expression in astrocytes [22], suggesting that modulation of glutamate clearance may contribute to SCFA-mediated neuroprotection.

Under pathological conditions of the nervous system, excessive elevation of extracellular glutamate causes overactivation of ionotropic glutamate receptors (particularly NMDARs), leading to massive calcium ion (Ca^2+^) influx, mitochondrial dysfunction, and increased ROS production, thereby triggering oxidative stress and neuronal injury [23,24]. In PD mouse models, membrane localization of EAAT2 is disrupted, leading to its intracellular redistribution and degradation [25,26]. Moreover, selective knockdown of astrocytic EAAT2 in the substantia nigra or striatum can induce molecular and behavioral changes reminiscent of PD, including neuronal loss, astrogliosis, abnormal gait, poorer motor performance, and depression-like features [27,28]. These observations suggest that SCFAs may reduce extracellular glutamate via modulation of EAAT2–mediated clearance, thereby lowering oxidative stress and conferring neuroprotection.

In the present study, we systematically evaluated the effects of SCFAs in a PD model by establishing four distinct experimental groups: a control group without any intervention to provide baseline data, a 1-methyl-4-phenyl-1,2,3,6-tetrahydropyridine (MPTP)-induced PD model group to recapitulate the core pathological and behavioral features of PD, an SCFA-treated group where PD model animals received SCFA administration to assess its potential therapeutic effects, and an SCFAs + dihydrokainic acid (DHK) cotreatment group—with DHK serving as an inhibitor of excitatory amino acid transporter 2 (EAAT2)—to investigate whether EAAT2 mediates the biological actions of SCFAs in PD. We found that SCFAs likely enhanced astrocytic EAAT2-mediated glutamate clearance, reduced glutamate-driven ROS production, and thereby activated the Nrf2 antioxidant pathway to alleviate neuronal injury and motor deficits. Interference with EAAT2 by DHK further demonstrated that EAAT2 function is critical for SCFA-mediated neuroprotection. Our results suggest that SCFAs regulate oxidative stress and promote neuronal survival in PD by enhancing EAAT2-mediated glutamate clearance, reducing ROS accumulation, and thereby restoring Nrf2-dependent antioxidant defenses—providing experimental support for SCFA-based therapeutic strategies.

## 2. Materials and Methods

### 2.1. Animals and Treatment

Male C57BL/6J mice (8 weeks old, 22–25 g) were purchased from Chongqing Ensiweier Experimental Animal Co., Ltd. (Chongqing, China). Animals were housed under standard laboratory conditions (25 ± 1 °C, 55 ± 10% humidity, 12 h light/dark cycle) with ad libitum access to food and water. All animal experiments were approved by the Ethics Committee of Chongqing Medical University and conducted in accordance with institutional guidelines.

Mice were randomly assigned to four groups (Control, MPTP, MPTP + SCFAs, MPTP + SCFAs + DHK; *n* = 12 per group, total *n* = 48) using a computer-generated random sequence. Cage positions were balanced across the room to minimize location effects, and behavioral tests were performed in a randomized order. Procedures were generally conducted at the same time of day to reduce potential variability due to circadian rhythms.

No inclusion or exclusion criteria were applied a priori. All animals completed the study without any deaths or extreme abnormalities. For each analysis, the exact number of animals per group was: Control, *n* = 12; MPTP, *n* = 12; MPTP + SCFAs, *n* = 12; MPTP + SCFAs + DHK, *n* = 12.

To induce Parkinson’s disease-like pathology, mice in the latter three groups received an intraperitoneal injection of 1-methyl-4-phenyl-1,2,3,6-tetrahydropyridine (MPTP) aqueous solution at a predetermined dose (30 mg/kg, MedChemExpress, Monmouth Junction, NJ, USA) for the first 7 consecutive days in the morning of each experimental day. On the same day, in the afternoon (with an interval of 6–8 h after MPTP injection to avoid potential drug interaction), mice in the SCFA-treated group were administered SCFA aqueous solution (butyrate, propionate, and acetate at a 1:1:1 weight ratio) via intragastric administration at a specified dose (300 mg/kg, MedChemExpress, Monmouth Junction, NJ, USA), while those in the SCFAs + DHK cotreatment group received sequential administration of DHK aqueous solution (10 mg/kg, MedChemExpress, Monmouth Junction, NJ, USA) followed by SCFAs (same dose and route as the SCFA-treated group) within 30 min. Both the SCFA-treated group and the SCFAs + DHK cotreatment group underwent the aforementioned drug administration protocol for 21 consecutive days.

During the whole experiment process, the control group received equal volumes of saline instead of MPTP, SCFAs, or DHK, following the same administration schedule (morning and afternoon intraperitoneally injections/intragastric administration) to maintain consistency. The MPTP-induced PD model group received an equal volume of saline (corresponding to the doses of SCFAs and DHK administered to the other treatment groups) in the afternoon. The SCFA-treated group received an intraperitoneal injection of saline at a volume equal to that of DHK administered to the SCFAs + DHK cotreatment group when SCFAs + DHK cotreatment group received DHK injection.

### 2.2. Behavioral Assessments

#### 2.2.1. Open Field Test (OFT)

Open field test: Mice were placed individually in a 50 × 50 × 40 cm square arena. Locomotor activity was recorded for 5 min using a video tracking system (Smart v3.0), and total distance traveled was measured.

#### 2.2.2. Pole Test

Pole test: Mice were placed head-upward on a vertical pole (50 cm height, 1 cm diameter). The time required to turn completely downward and descend to the base was recorded.

#### 2.2.3. Rotarod Test

Rotarod test: Mice were trained to stay on a rotating rod (4–40 rpm) (Sansbio, Shanghai, China) for 3 consecutive days before testing. The latency to fall was recorded, and the average of three trials was calculated.

#### 2.2.4. Grip Strength Test

Grip strength test: Forelimb grip strength was measured using a grip strength meter (Sansbio, Shanghai, China). Each mouse was tested three times, and the mean value was used for analysis.

### 2.3. Tissue Preparation

The substantia nigra and striatum were isolated using a standardized microdissection protocol. Freshly harvested brain tissue was positioned ventral side up on an ice-cold glass slide, with all procedures maintained at 4 °C in an ice bath to pre-vent tissue autolysis. The midbrain region was identified with reference to the mouse brain atlas, and a 2–3 mm thick tissue block was excised along the anterior and posterior margins of the midbrain using ophthalmic scissors. Under a dissecting microscope, extraneous regions were carefully removed to retain only the core midbrain and striatal structures. Fine ophthalmic forceps were then used to gently separate the cerebral peduncle tissue on the ventral aspect of the midbrain, exposing the grayish-black substantia nigra, which includes both the pars compacta (SNc) and pars reticulata (SNr). The substantia nigra was meticulously dissected along its natural anatomical boundaries to avoid traction or compression-induced tissue damage. The isolated substantia nigra and striatum were transferred to pre-chilled physiological saline for rinsing to remove residual contaminants before immediate processing for subsequent biochemical assays or protein extraction.

### 2.4. Western Blot Analysis

Striatal tissues from 3 mice per group were homogenized in radioimmunoprecipitation assay (RIPA) lysis buffer containing protease and phosphatase inhibitors (Beyotime, Shanghai, China) and centrifuged at 12,000× *g* for 10 min at 4 °C. The supernatants were collected, and protein concentrations were determined using a bicinchoninic acid (BCA) protein assay kit (Epizyme, Shanghai, China).

Equal amounts of protein (30 µg) were separated by sodium dodecyl sulfate-polyacrylamide gel (SDS-PAGE) and transferred to polyvinylidene fluoride (PVDF) membranes (Epizyme, Shanghai, China). Membranes were blocked with 5% non-fat milk for 1.5 h and incubated overnight at 4 °C with primary antibodies against TH, Nrf2 (Proteintech, Wuhan, China), EAAT2 (Abmart, Shanghai, China, diluted 1:500), HO-1 (Abmart, Shanghai, China, diluted 1:1000), SOD1 (Abmart, Shanghai, China, diluted 1:1500), and SOD2 (Abmart, Shanghai, China, diluted 1:1000). After washing, membranes were incubated with horseradish peroxidase-conjugated (HRP-conjugated) secondary antibodies for 1.5 h at room temperature. Protein bands were visualized using an enhanced chemiluminescence (ECL) kit (Epizyme, Shanghai, China) and quantified by ImageJ (version 1.53t, NIH, USA). β-Actin (Proteintech, Wuhan, China, diluted 1:20,000) was used as the internal control.

### 2.5. Immunohistochemistry

Immunohistochemistry (IHC) was performed to detect TH in the substantia nigra and glial fibrillary acidic protein (GFAP) in the striatum. Brain tissues from 3 mice per group were used for TH staining in the substantia nigra, and from 3 mice per group for GFAP staining in the striatum. Tissues were fixed in 4% paraformaldehyde, dehydrated, paraffin-embedded, and cut into 5 µm sections. After deparaffinization and antigen retrieval in ethylenediaminetetraacetic acid (EDTA), sections were incubated with 3% hydrogen peroxide to quench endogenous peroxidase activity, blocked with 5% bovine serum albumin (BSA), and incubated overnight at 4 °C with primary antibodies against TH (1:500, Proteintech, Wuhan, China) and GFAP (1:3200, Cell Signaling Technology, Danvers, MA, USA). Sections were then incubated with HRP-conjugated secondary antibodies using an IHC kit (Proteintech, Wuhan, China) according to the manufacturer’s protocol. Immunoreactive signals were visualized with a diaminobenzidine (DAB) chromogenic substrate and counterstained with hematoxylin. In this study, three animals were selected per group, with one tissue section obtained from each animal. All sections were fully scanned using the Smart Digital Slide Scanner (UISCAN, Shanghai, China), and the exposure parameters were kept consistent across all images. One visual field was randomly selected from each section and magnified 5–10 times for image acquisition. The images presented for the striatum were acquired from a standardized field of view positioned in the central region of the structure This specific location was selected to ensure consistent sampling across all animals and to avoid potential heterogeneity near striatal borders, thereby providing a representative assessment of the entire structure. Subsequently, ImageJ (version 1.53t, NIH, USA) was used to quantitatively analyze the percentage of positive area (%Area). The specific procedure is as follows: all images were uniformly converted to 8-bit grayscale format, and a consistent threshold was set to identify positive signal regions, followed by the measurement of parameters of the target regions—with the positive area ratio serving as the key evaluation metric. All image processing and quantitative analyses were performed in a blinded manner, where operators were completely unaware of the experimental grouping.

### 2.6. Biochemical Assays

The levels of glutathione (GSH), malondialdehyde (MDA), superoxide dismutase (SOD), and glutamate in the substantia nigra were determined using commercial biochemical kits purchased from Solarbio Science & Technology Co., Ltd. (Beijing, China), according to the manufacturer’s instructions. Striatal tissues from 6 mice per group were used for these biochemical assays. The tissues were homogenized in ice-cold PBS (reagents provided in the kit) and centrifuged at 10,000× *g* for 10 min at 4 °C. The supernatants were used for biochemical detection. GSH content was measured by the 5,5′-dithiobis (2-nitrobenzoic acid) (DTNB) colorimetric method, MDA content by the thiobarbituric acid (TBA) reaction, SOD activity by the WST-1 method, and glutamate concentration by enzymatic oxidation at 340 nm.

### 2.7. Enzyme-Linked Immunosorbent Assay (ELISA)

Inflammatory cytokines IL-1β, IL-6, and TNF-α were measured in plasma from 6 mice per group using mouse ELISA kits (Proteintech, Wuhan, China) following the manufacturer’s instructions. Absorbance was read at 450 nm using a microplate reader (Thermo Fisher Scientific, Waltham, MA, USA), and cytokine concentrations were calculated based on standard curves.

### 2.8. Statistical Analysis

All experimental data are presented as the mean ± standard error of the mean (SEM). Statistical analyses were performed using GraphPad Prism 9.0 (GraphPad Software, San Diego, CA, USA). Data were analyzed by investigators blinded to the group assignments. Comparisons among multiple groups were conducted using one-way analysis of variance (ANOVA) followed by Tukey’s post hoc tests. Assumptions of normality and homogeneity of variance were assessed, and no violations were observed. Differences were considered statistically significant at *p* < 0.05.

## 3. Results

### 3.1. Monitoring of Body Weight Changes Before and During the Experiment

**Figure 1:**
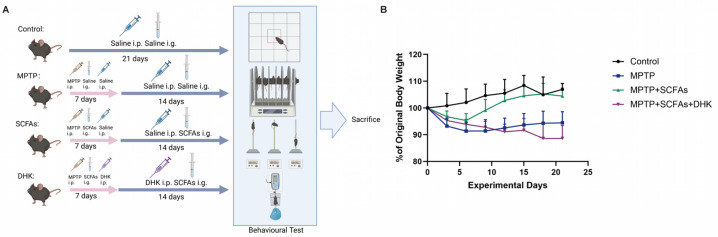
The experimental process and the body weight changes of mice. (**A**) Schematic diagram of the experimental process; (**B**) The body weight changes of mice over 21 days. MPTP, 1-methyl-4-phenyl-1,2,3,6-tetrahydropyridine; SCFAs, short-chain fatty acids; DHK, dihydrokainic acid. i.p., intraperitoneal; i.g., intragastric. Created in BioRender. https://BioRender.com/y3iiu01 (accessed on 15 November 2025).

Body weight changes were monitored before (day 0) and during the experimental period (day 21). Compared with the control group, mice treated with MPTP exhibited a continuous decline in body weight during administration and a slower subsequent increase, indicating that MPTP treatment restricted normal weight gain. SCFA administration ameliorated this condition, whereas DHK treatment partially maintained the downward trend (Figure 1B).

### 3.2. SCFA Treatment Ameliorates Motor Deficits in MPTP-Induced Parkinson’s Disease Mice

**Figure 2:**
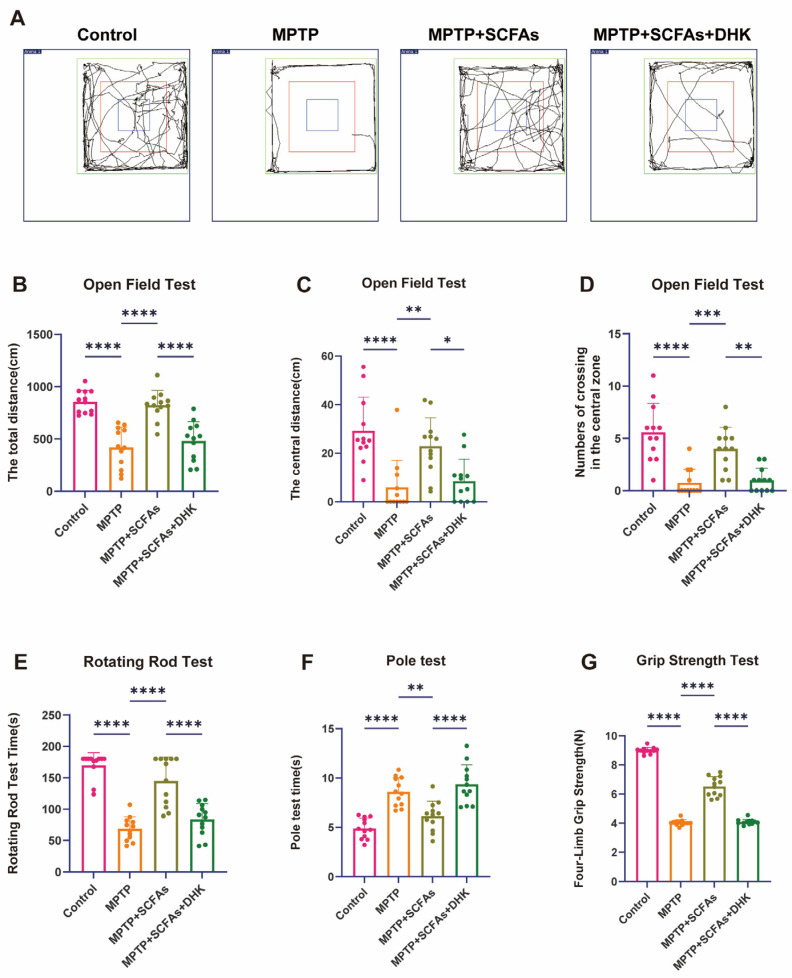
Behavioral test results. (**A**) Representative locomotion traces in the Open Field Test among Control, MPTP, MPTP + SCFAs, and MPTP + SCFAs + DHK groups; (**B**) Open Field Test result of the total distance traveled (in centimeters) by mice in different groups; (**C**) Open Field Test result of the distance traveled in the central zone (in centimeters) among the groups; (**D**) Open Field Test result of the number of crossings in the central zone for each group; (**E**) Rotating Rod Test results showing the rotating rod test times in different groups; (**F**) Pole Test results presenting the pole test times; (**G**) Grip Strength Test results indicating the four-limb grip strength (in Newtons) for each group. Data are presented as mean ± SEM (*n* = 12 per group). * *p* < 0.05, ** *p* < 0.01, *** *p* < 0.001, **** *p* < 0.0001 vs. indicated groups. MPTP, 1-methyl-4-phenyl-1,2,3,6-tetrahydropyridine; SCFAs, short-chain fatty acids; DHK, dihydrokainic acid.

Behavioral tests were conducted to evaluate the effects of SCFAs on MPTP-induced motor impairments. In the open field test, the total distance traveled was significantly reduced in MPTP-treated mice compared with controls (*p* < 0.0001), indicating impaired locomotor activity. SCFA administration significantly increased total distance (*p* < 0.0001), whereas DHK pretreatment partially abolished this improvement (*p* < 0.0001). Similarly, the distance traveled in the central zone and the number of entries into the central zone were markedly decreased in MPTP-treated mice compared with controls (both *p* < 0.0001), reflecting enhanced anxiety-like behavior. SCFA treatment significantly increased both central zone distance (*p* < 0.01) and central entries (*p* < 0.001), while DHK pretreatment attenuated these effects (central zone distance: *p* < 0.05; central entries: *p* < 0.01) (Figure 2A–D).

In the rotating rod test, MPTP exposure decreased the latency to fall (*p* < 0.0001), which was restored by SCFA intervention (*p* < 0.0001). DHK pretreatment significantly reduced the SCFA-induced improvement (*p* < 0.0001, Figure 2E). In the pole test, MPTP mice exhibited prolonged descent times (*p* < 0.0001), which were significantly shortened after SCFA treatment (*p* < 0.01). This improvement was partially abolished by DHK pretreatment (*p* < 0.0001) (Figure 2F). Additionally, grip strength tests confirmed that SCFAs enhanced MPTP-impaired muscle strength (*p* < 0.0001, Figure 2D), whereas DHK administration attenuated this enhancement (*p* < 0.0001). These results indicate that SCFA treatment significantly alleviates MPTP-induced motor and anxiety-like deficits in mice, whereas DHK pretreatment partially reverses these beneficial effects, suggesting that EAAT2 activity is essential for the neuroprotective actions of SCFAs.

### 3.3. SCFAs Increase Dopaminergic Neuron Survival

**Figure 3:**
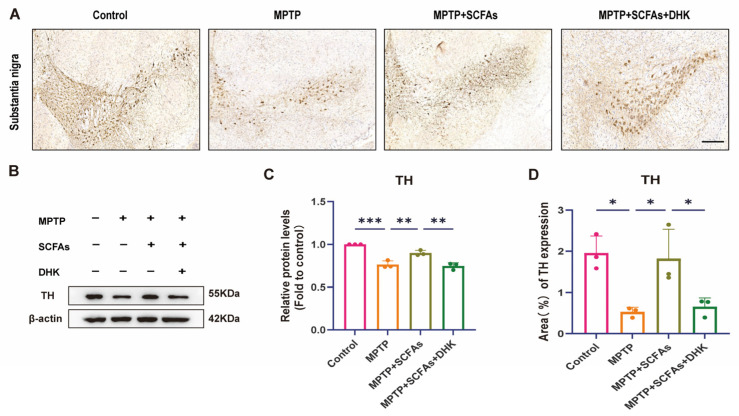
Immunohistochemical staining (to assess TH-positive neurons in the substantia nigra) and Western blotting (to quantify TH protein levels in the striatum). (**A**) Representative images of TH-positive neurons in the substantia nigra among Control, MPTP, MPTP + SCFAs, and MPTP + SCFAs + DHK groups, Scale bar: 200 µm; (**B**) Western blot analysis of TH protein levels (with β-actin as a loading control) in the striatum among different groups; (**C**) Quantification of relative TH protein levels (fold to control) across different groups; (**D**) Quantification of the area percentage of TH expression in the substantia nigra among the groups. Data are presented as mean ± SEM (*n* = 3 per group). * *p* < 0.05, ** *p* < 0.01, *** *p* < 0.001 vs. indicated groups. MPTP, 1-methyl-4-phenyl-1,2,3,6-tetrahydropyridine; SCFAs, short-chain fatty acids; DHK, dihydrokainic acid; TH, tyrosine hydroxylase.

To evaluate the integrity of dopaminergic neurons, TH immunohistochemistry and Western blot analysis of TH protein levels were performed. Compared with the control group, MPTP exposure significantly reduced the density of TH-positive neurons in the substantia nigra (*p* < 0.05). SCFA treatment markedly preserved TH-positive neurons (*p* < 0.05), whereas this neuroprotective effect was partially attenuated when co-administered with DHK (*p* < 0.05) (Figure 3A,D). Consistently, Western blot results showed that TH protein levels in the striatum were significantly decreased in the MPTP group compared with controls (*p* < 0.001), while SCFA administration significantly increased TH expression (*p* < 0.01); this improvement was notably diminished by DHK pretreatment (*p* < 0.01) (Figure 3B,C). These findings indicate that SCFAs protect dopaminergic neurons by enhancing striatal TH expression and mitigating neuronal loss, and that DHK pretreatment partially abolishes these protective effects, suggesting a mechanism partially dependent on EAAT2.

### 3.4. SCFAs Attenuate MPTP-Induced Neuroinflammation

**Figure 4:**
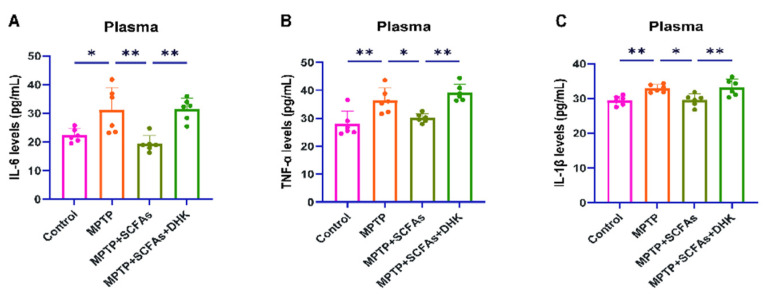
Enzyme-linked immunosorbent assay (ELISA) (to measure plasma levels of IL-6, TNF-α, and IL-1β) results. (**A**) Levels of IL-6 in plasma (pg/mL) among Control, MPTP, MPTP + SCFAs, and MPTP + SCFAs + DHK groups; (**B**) TNF-α in plasma (pg/mL) across different groups; (**C**) Levels of IL-1β in plasma (pg/mL) for each group. Data are presented as mean ± SEM (*n* = 6 per group). * *p* < 0.05, ** *p* < 0.01 vs. indicated groups. MPTP, 1-methyl-4-phenyl-1,2,3,6-tetrahydropyridine; SCFAs, short-chain fatty acids; DHK, dihydrokainic acid; IL-6, interleukin-6; TNF-α, tumor necrosis factor-α; IL-1β, interleukin-1β.

Plasma cytokine analysis revealed that MPTP treatment significantly elevated serum levels of inflammatory markers, including IL-6 (*p* < 0.05), TNF-α (*p* < 0.01), and IL-1β (*p* < 0.01). Administration of SCFAs markedly suppressed these increases, reducing IL-6 (*p* < 0.05), TNF-α (*p* < 0.01), and IL-1β (*p* < 0.01). Notably, combined treatment with DHK and SCFAs partially reversed the anti-inflammatory effects of SCFAs, as plasma cytokine levels were significantly higher than those in the SCFA-only group (*p* < 0.05) (Figure 4A–C).

### 3.5. SCFAs Reduce Astrocyte Activation, Increase EAAT2, and Promote Glutamate Clearance in PD

**Figure 5:**
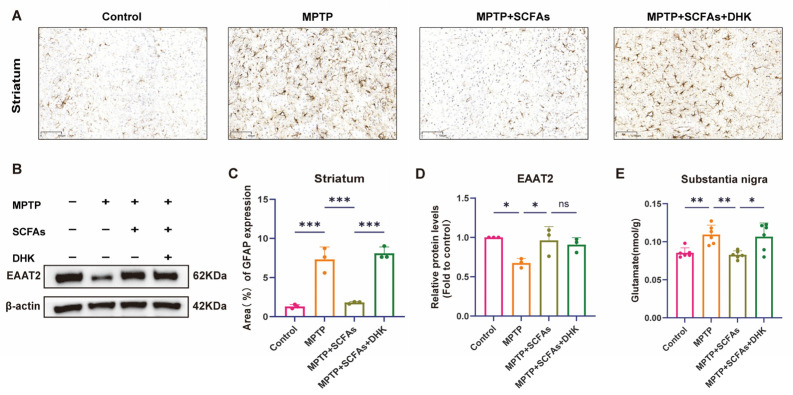
Immunohistochemical staining, Western blotting, and biochemical quantification to explore the effects of MPTP, SCFAs, and DHK on GFAP expression in the striatum, EAAT2 protein levels, and glutamate levels in the substantia nigra of mice. (**A**) Representative images of GFAP staining in the striatum among Control, MPTP, MPTP + SCFAs, and MPTP + SCFAs + DHK groups; (**B**) Western blot analysis of EAAT2 protein levels (with β-actin as a loading control) in different groups; (**C**) Quantification of the area percentage of GFAP expression in the striatum across each group; (**D**) Quantification of relative EAAT2 protein levels (fold to control) among the groups; (**E**) Levels of glutamate (nmol/g) in the substantia nigra (SN) for each group. Data are presented as mean ± SEM (*n* = 3–6 per group). * *p* < 0.05, ** *p* < 0.01, *** *p* < 0.001 vs. indicated groups; ns = not significant. MPTP, 1-methyl-4-phenyl-1,2,3,6-tetrahydropyridine; SCFAs, short-chain fatty acids; DHK, dihydrokainic acid; GFAP, glial fibrillary acidic protein; EAAT2, excitatory amino transporter 2.

GFAP immunohistochemical staining further indicated that MPTP induced marked astrocytic activation, which was significantly reduced by SCFA treatment (*p* < 0.001). DHK pretreatment partially reversed this reduction (*p* < 0.001) (Figure 5A,C). Consistently, Western blot analysis showed that MPTP administration significantly downregulated EAAT2 protein expression in the striatum (*p* < 0.05). SCFA treatment significantly restored EAAT2 expression (*p* < 0.05), whereas the functional inhibitor DHK did not suppress EAAT2 expression (Figure 5B,D). Measurement of glutamate concentration in the substantia nigra revealed a significant increase in extracellular glutamate levels in MPTP-treated mice (*p* < 0.01). SCFA treatment effectively reduced this concentration (*p* < 0.01), while DHK administration counteracted this beneficial effect (*p* < 0.05) (Figure 5E). These findings indicate that SCFAs alleviate striatal astrocyte hyperactivation and excitotoxic stress by promoting EAAT2-mediated glutamate clearance.

### 3.6. SCFAs Enhance Antioxidant Capacity and Mitigate Oxidative Stress

**Figure 6:**
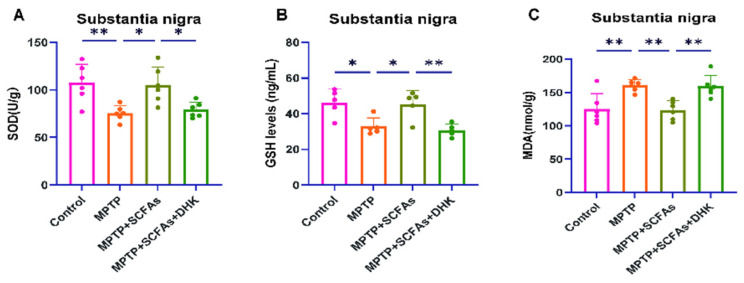
Effects of SCFAs and DHK on antioxidant capacity in the substantia nigra of MPTP-treated mice. (**A**) SOD activity, (**B**) GSH levels, and (**C**) MDA levels in the substantia nigra. Data are presented as mean ± SEM (*n* = 6 per group). * *p* < 0.05, ** *p* < 0.01 vs. indicated groups. MPTP, 1-methyl-4-phenyl-1,2,3,6-tetrahydropyridine; SCFAs, short-chain fatty acids; DHK, dihydrokainic acid; GSH, Glutathione; MDA, malondialdehyde; SOD, superoxide dismutase.

Biochemical assays consistently showed that MPTP exposure significantly decreased GSH content (*p* < 0.05) and SOD activity (*p* < 0.01), while concomitantly increasing MDA levels (*p* < 0.01). Administration of SCFAs increased GSH content and SOD activity (both *p* < 0.05) and decreased MDA levels (*p* < 0.01), whereas DHK partially blocked these effects, resulting in reduced GSH content and SOD activity (both *p* < 0.01) and elevated MDA levels (*p* < 0.05) (Figure 6A–C). These results indicate that SCFAs enhance antioxidant capacity and alleviate oxidative stress, a mechanism that may involve EAAT2.

### 3.7. SCFAs Restore Antioxidant Protein Expression via EAAT2-Dependent Nrf2 Activation

**Figure 7:**
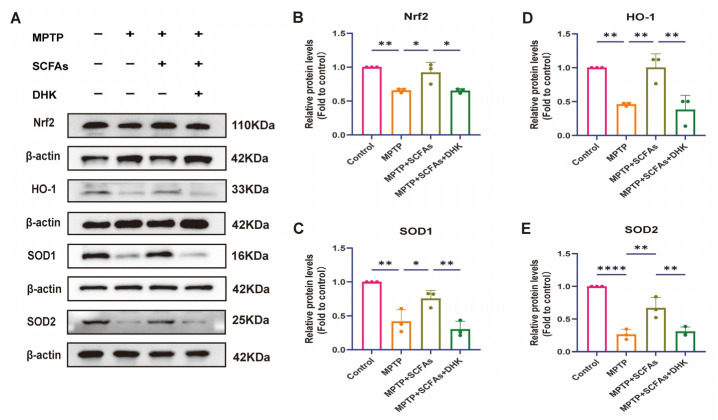
Effects of SCFAs and DHK on oxidative stress-related protein expression. (**A**) Representative Western blots showing the expression of Nrf2, HO-1, SOD1, and SOD2 with β-actin as the loading control. Relative protein levels of (**B**) Nrf2, (**C**) SOD1, (**D**) HO-1, and (**E**) SOD2 quantified by densitometry and normalized to control. Data are presented as mean ± SEM (*n* = 3 per group). * *p* < 0.05, ** *p* < 0.01, **** *p* < 0.0001 vs. indicated groups. MPTP, 1-methyl-4-phenyl-1,2,3,6-tetrahydropyridine; SCFAs, short-chain fatty acids; DHK, dihydrokainic acid; Nrf2, nuclear factor-erythroid 2 related factor; HO-1, heme oxygenase 1; SOD1, superoxide dismutase 1; SOD2, superoxide dismutase 2.

To further examine changes in redox homeostasis, we used Western blotting to assess the expression of canonical oxidative stress-related proteins in the striatum. MPTP treatment significantly reduced the protein levels of SOD1 (*p* < 0.01), SOD2 (*p* < 0.0001), Nrf2 and HO-1 (both *p* < 0.01), indicating pronounced oxidative damage in PD mice. SCFA intervention markedly reversed these decreases—SOD1 (*p* < 0.05), SOD2 (*p* < 0.01), Nrf2 (*p* < 0.05) and HO-1 (*p* < 0.01)—whereas DHK pretreatment partially attenuated the SCFA-mediated restoration: SOD1 (*p* < 0.01), SOD2 (*p* < 0.01), Nrf2 (*p* < 0.05) and HO-1 (*p* < 0.01) (Figure 7A–E). These findings indicate that SCFAs alleviate oxidative stress primarily by restoring Nrf2-dependent antioxidant defenses, an effect mediated, at least in part, through EAAT2-driven regulation of glutamate homeostasis.

## 4. Discussion

Parkinson’s disease is a progressive neurodegenerative disorder characterized by dopaminergic neuronal loss and motor impairment [3,29]. Consistent with established PD models [30,31,32], MPTP-treated mice in our study exhibited bradykinesia, impaired locomotion, and reduced grip strength. SCFA administration significantly ameliorated these deficits, in agreement with previous findings showing that butyrate alone improves motor performance in MPTP mice [33]. The improvement in grip strength further suggests that SCFAs not only enhance motor coordination but may also mitigate neuromuscular impairment. In parallel, SCFAs preserved TH expression in both the substantia nigra and striatum, supporting a neuroprotective role consistent with prior studies [29]. To determine whether EAAT2 contributes to these effects, we employed DHK, a selective EAAT2 inhibitor [34].

DHK markedly attenuated the SCFA-induced improvements in motor behavior and abolished the preservation of TH expression. These findings indicate that EAAT2 activity is a key mediator of the beneficial effects of SCFAs on dopaminergic neurons. In PD and other neuroinflammatory conditions, reactive astrocytes exhibit reduced EAAT2 expression or function, impairing glutamate clearance and exacerbating excitotoxic injury [21,35,36,37,38,39,40,41]. In our study, SCFAs suppressed astrocyte activation, as evidenced by reduced GFAP immunoreactivity, whereas DHK reversed this effect. Because GFAP upregulation is a hallmark of reactive astrogliosis and reflects a shift toward a pro-inflammatory astrocytic state, its normalization by SCFAs suggests that enhanced EAAT2-mediated glutamate clearance helps limit astrocyte reactivity. Although DHK did not significantly alter EAAT2 protein abundance, glutamate transport assays confirmed potent inhibition of transporter function, indicating that DHK primarily affects EAAT2 activity rather than expression.

Neuroinflammation is another key contributor to PD pathology [42]. Consistent with previous reports [18], SCFA treatment significantly reduced circulating levels of IL-6, TNF-α, and IL-1β, whereas DHK abolished this anti-inflammatory effect. Notably, DHK alone promoted astrocyte polarization, which is known to induce the release of pro-inflammatory mediators such as TNF-α, IL-1α, C1q, and complement component C3 [43,44,45]. These findings suggest that loss of EAAT2 activity may indirectly enhance neuroinflammation by facilitating astrocytic activation.

Oxidative stress constitutes a major pathological driver in PD [46,47,48,49,50]. In the substantia nigra, SCFAs restored GSH levels, reduced MDA accumulation, and preserved SOD activity, indicating a robust antioxidative effect consistent with earlier studies [19,51]. SCFAs also markedly increased nuclear Nrf2 and elevated downstream antioxidant enzymes including HO-1, SOD1, and SOD2 [52,53,54,55,56,57,58]. Importantly, these effects were reversed by DHK, suggesting that EAAT2-dependent glutamate clearance may reduce ROS accumulation and thereby facilitate Nrf2 activation. Given that excess glutamate induces mitochondrial dysfunction and oxidative stress, and that ROS can in turn suppress Nrf2 signaling [59,60], our results support a model in which SCFAs reduce excitotoxicity and oxidative damage through EAAT2-mediated regulation of glutamate homeostasis.

Emerging evidence suggests that SCFAs exert regulatory effects on astrocytes that may help explain the EAAT2 elevation observed in our MPTP model. Sun et al. reported that long-term SCFA supplementation enhances the astrocyte–neuron glutamate–glutamine shuttle in Amyloid Precursor Protein/Presenilin-1 (APP/PS1) mice, and scRNA-seq analysis showed increased astrocytic expression of EAAT2 following SCFA treatment [61]. Similarly, Spichak et al. demonstrated that microbially derived SCFAs markedly reshape astrocyte transcriptional programs, including genes involved in glutamate metabolism and redox regulation [62]. These findings support the plausibility that SCFAs promote a glutamate-handling phenotype in astrocytes, consistent with our observation that SCFAs restore EAAT2 levels and reduce striatal glutamate accumulation.

Epigenetic and receptor-mediated signaling mechanisms may further contribute to the SCFA-induced increase in EAAT2. EAAT2 expression is strongly influenced by histone acetylation, and multiple Histone Deacetylase (HDAC) inhibitors have been shown to enhance EAAT2 transcription and protein levels in astrocytes [63]. Notably, sodium butyrate—a major SCFA and HDAC inhibitor—reversed manganese-induced reductions in EAAT2 and improved motor deficits in vivo, directly linking SCFA to restoration of astrocytic glutamate transporters [22]. Although these mechanisms are biologically plausible and supported by published work, we acknowledge that our study did not directly test upstream SCFA receptors, histone acetylation changes, or astrocyte-specific transcriptomic responses. Thus, the SCFA–EAAT2–glutamate–ROS axis proposed here should be viewed as a hypothesis-generating model, and future studies employing receptor antagonists, chromatin assays, and astrocyte-targeted omics will be needed to confirm the precise molecular pathways involved. In our study we selected a total SCFA dose of 300 mg/kg (acetate: propionate: butyrate = 1:1:1) as a moderate, neuroprotective range based on previous PD and PD-like rodent studies in which sodium butyrate or SCFA treatments between 150 and 600 mg/kg/day improved motor behavior, preserved nigrostriatal dopaminergic neurons, and reduced neuroinflammation without obvious toxicity [33,64,65,66]. Consistent with this literature, we aimed to use a dose that is clearly within the effective window reported for butyrate-based interventions in PD models while avoiding the very high “pharmacological” exposures that have been associated with stress-like or toxic responses in non-PD settings, thereby balancing efficacy and safety for chronic administration [67,68]. Notably, doses around 1200 mg/kg i.p. have been used mainly to probe histone-deacetylase-related mechanisms and are now recognized to trigger a robust hypothalamic–pituitary–adrenal stress response and reversible metabolic disturbances, so we deliberately avoided this super-pharmacological range when defining a physiologically relevant SCFA dose for PD [68]. Furthermore, we explicitly acknowledge that determining the dose–response characteristics of SCFAs remain an important next step, and future work should systematically examine different dosing regimens to define the optimal therapeutic window and pharmacological profile of SCFAs in PD models.

## 5. Conclusions

**Figure 8:**
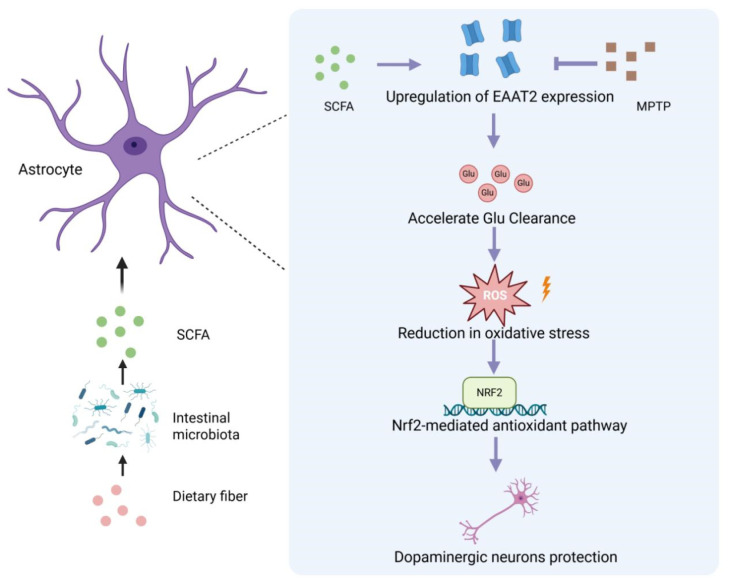
Mechanistic illustration of SCFA-mediated neuroprotection via astrocytic EAAT2 in Parkinson’s disease. Dietary fiber is metabolized by the intestinal microbiota to produce short-chain fatty acids (SCFAs), which act on astrocytes to upregulate EAAT2 expression. Enhanced EAAT2 function accelerates glutamate (Glu) clearance, reducing extracellular Glu-induced reactive oxygen species (ROS) accumulation. This decrease in oxidative stress activates the Nrf2-mediated antioxidant pathway, ultimately contributing to the protection of dopaminergic neurons against MPTP-induced neurotoxicity. Created in BioRender. https://BioRender.com/nqqr282 (accessed on 2 November 2025).

In summary, our study demonstrates that SCFAs exert multifaceted neuroprotective effects in an MPTP-induced PD model. By enhancing astrocytic EAAT2-mediated glutamate clearance, SCFAs reduce excitotoxicity, suppress astrocyte activation, and ultimately preserve dopaminergic neurons and motor function. In parallel, SCFAs restore redox homeostasis by decreasing ROS accumulation and promoting Nrf2-dependent antioxidant defenses. The reversal of these effects by the EAAT2 inhibitor DHK underscores the central role of EAAT2 in mediating SCFA-induced neuroprotection. Together, these findings provide mechanistic insight into how SCFAs modulate glutamate and oxidative pathways and highlight EAAT2 as a promising therapeutic target. Future studies evaluating dose–response relationships and delineating upstream molecular signaling will be critical for advancing SCFA-based interventions for PD (Figure 8).

## Data Availability

Our data has other research purposes and is not readily available to the public.

## Abbreviations

The following abbreviations are used in this manuscript:

AMPA	α-amino-3-hydroxy-5-methyl-4-isoxazolepropionic acid
BCA	Bicinchoninic acid
C1q	Complement component 1q
DHK	Dihydrokainic acid
DTNB	5,5′-Dithiobis(2-nitrobenzoic acid)
EAATs	Excitatory amino acid transporters
ECL	Enhanced chemiluminescence
ELISA	Enzyme-linked immunosorbent assay
GFAP	Glial fibrillary acidic protein
Glu	Glutamate
HO-1	Heme oxygenase-1
IHC	Immunohistochemistry
IL-1α	Interleukin-1 alpha
IL-1β	Interleukin-1 beta
IL-6	Interleukin-6
MDA	Malondialdehyde
MPTP	1-Methyl-4-phenyl-1,2,3,6-tetrahydropyridine
NMDA	N-methyl-D-aspartate
NMDARs	N-methyl-D-aspartate receptors
Nrf2	Nuclear factor-erythroid 2–related factor 2
OFT	Open field test
PBS	Phosphate-buffered saline
PD	Parkinson’s disease
PVDF	Polyvinylidene fluoride
ROS	Reactive oxygen species
SCFAs	Short-chain fatty acids
SOD1/2	Superoxide dismutase 1/2
TBA	Thiobarbituric acid
TH	Tyrosine hydroxylase
TNF-α	Tumor necrosis factor-alpha
WST-1	Water-soluble tetrazolium salt-1
